# Features of alternative splicing in stomach adenocarcinoma and their clinical implication: a research based on massive sequencing data

**DOI:** 10.1186/s12864-020-06997-x

**Published:** 2020-08-24

**Authors:** Yuanyuan Zhang, Shengling Ma, Qian Niu, Yun Han, Xingyu Liu, Jie Jiang, Simiao Chen, Haolong Lin

**Affiliations:** 1 Department of Oncology, Tongji Hospital, Tongji Medical College, Huazhong University of Science and Technology, Wuhan, 430030 China; 2 Institute of Hematology, Union Hospital, Tongji Medical College, Huazhong University of Science and Technology, Wuhan, China; 3 Department of Ophthalmology, Tongji Hospital, Tongji Medical College, Huazhong University of Science and Technology, Wuhan, 430030 China; 4 Department of Gynecology and Obstetrics, Tongji Hospital, Tongji Medical College, Huazhong University of Science and Technology, Wuhan, 430030 China; 5 Department of Anesthesiology, Tongji Hospital, Tongji Medical College, Huazhong University of Science and Technology, Wuhan, 430030 China; 6 Department of Neurology, Tongji Hospital, Tongji Medical College, Huazhong University of Science and Technology, Wuhan, 430030 China; 7 Department of Hematology, Tongji Hospital, Tongji Medical College, Huazhong University of Science and Technology, Wuhan, 430030 China

**Keywords:** Bioinformatic analysis, Alternative splicing, Stomach adenocarcinoma, Survival, Splicing factor, Prognosis

## Abstract

**Background:**

Alternative splicing (AS) offers a main mechanism to form protein polymorphism. A growing body of evidence indicates the correlation between splicing disorders and carcinoma. Nevertheless, an overall analysis of AS signatures in stomach adenocarcinoma (STAD) is absent and urgently needed.

**Results:**

2042 splicing events were confirmed as prognostic molecular events. Furthermore, the final prognostic signature constructed by 10 AS events gave good result with an area under the curve (AUC) of receiver operating characteristic (ROC) curve up to 0.902 for 5 years, showing high potency in predicting patient outcome. We built the splicing regulatory network to show the internal regulation mechanism of splicing events in STAD. QKI may play a significant part in the prognosis induced by splicing events.

**Conclusions:**

In our study, a high-efficiency prognostic prediction model was built for STAD patients, and the results showed that AS events could become potential prognostic biomarkers for STAD. Meanwhile, QKI may become an important target for drug design in the future.

## Background

Gastric cancer (GC) is the fourth major cancer threat to human health in the world whose etiology remains unclear, with 989,000 new cases and 738,000 deaths every year [1]. Most (about 90%) of gastric cancers are adenocarcinomas, which originate from the epithelial cells in the most superficial layer of the gastric wall and are caused by malignant changes in gastric gland cells. Although significant progress has been made in the study of epidemiology, pathological mechanisms and treatment options, the medical burden still exceeds expectations [2]. It is not optimistic that complete surgical resection is still the only solution for doctors to treat gastric cancer [3, 4]. The widespread implementation and application of adjuvant and neoadjuvant therapy have increased the 5-year overall survival rate by 10–15%, but it is worth thinking that there is no global consensus on the best treatment scheme [2]. Therefore, it is urgent to explore new and accurate biomarkers to evaluate the diagnosis and prognosis of STAD patients.

Eukaryotic cells produce various regulatory changes and perform complex functions to adapt to changes in the environment, largely due to the diversity of proteins. A common mechanism is that a limited number of gene sets produce a large number of mRNA isoforms through alternative splicing of pre-mRNA [5]. Alternative splicing actually regulates gene expression at the intron/exon level [6–8]. In addition, alternative splicing causes the premature occurrence of termination codon in mRNA, which degrades immediately upon discovery to prevent its translation [9]. Therefore, alternative splicing is a key biological process in cells, and different mRNA splicing isoforms make the final protein products perform different functions.

More and more studies have found that splicing disorder can be used as a marker of tumor development [10] and as a key mechanism involved in the broad biological process of cancer [11, 12]. It is noteworthy that some important splicing factors can change the alternative splicing mode of target genes, thus forming a favorable environment for promoting the occurrence and development of cancer [13]. In general, comprehensive and in-depth analysis of alternative splicing can dig out potential biomarkers of malignant tumors, so as to assist physicians in clinical diagnosis and prognosis judgment [12, 14].

We used a variety of bioinformatics analysis methods to explore prognostic factors in STAD. COX regression analysis helped us screen out significant prognostic markers for further study. According to the regulatory relationship between AS events and splicing factors in STAD, a clear network diagram was drawn to find out the potential mechanism. These results provide a basic direction for further exploration of the molecular mechanism and diagnostic markers of STAD.

## Results

### Survival associated AS events

**Fig. 1:**
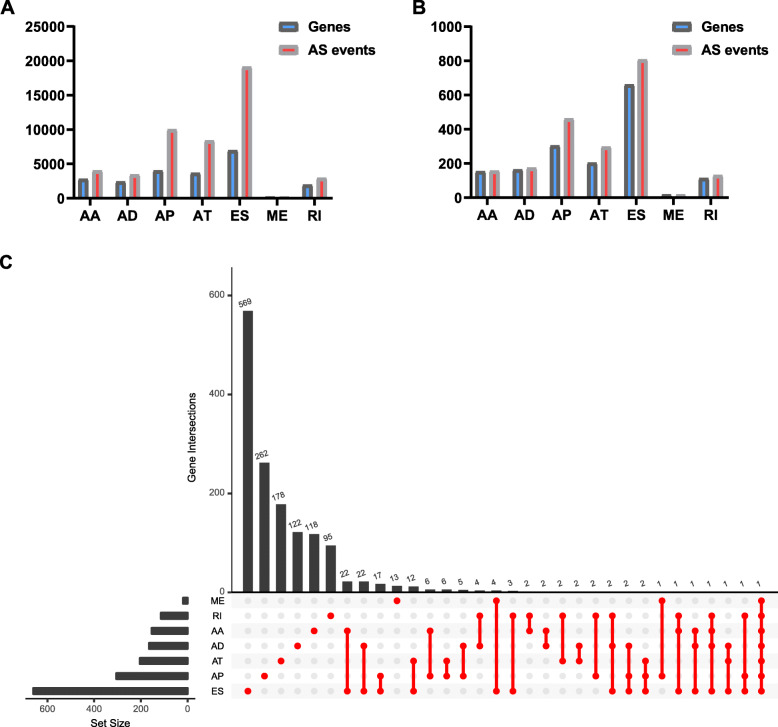
Prognosis-related alternative splicing (AS) events. **a** The number of AS events and corresponding genes included in the present study; **b** The number of prognosis-related AS events and corresponding genes obtained by using univariate COX analysis; **c** UpSet plot of interactions between the seven types of survival associated AS events in STAD. One gene may have up to seven types of alternative splicing to be associated with patient survival

As a whole, there are 4006 AA events in 2799 genes, 3450 AD events in 2401 genes, 10,004 AP events in 4025 genes, 8390 AT events in 3666 genes, 19,121 ES events in 6973 genes, 226 ME events in 219 genes, and 2944 RI events in 1956 genes for evaluation of prognostic value (Fig. 1a). The initial clinical data downloaded from the TCGA website is in the supplementary files (Additional file 1). A total of 157 AA events in 153 genes, 174 AD events in 164 genes, 461 AP events in 304 genes, 297 AT events in 203 genes, 805 ES events in 660 genes, 18 ME events in 18 genes, and 130 RI events in 113 genes were identified as prognostic AS events (*P* < 0.05) (Fig. 1b, Additional file 2). Thus, one gene might have two or more AS events that were markedly related to the survival of STAD patients. The ES which was vividly revealed by the UpSet plot was the most common prognosis-related event, and a gene could have up to seven prognosis-related events (Fig. 1c).

### Molecular characteristics of survival related AS events

**Fig. 2:**
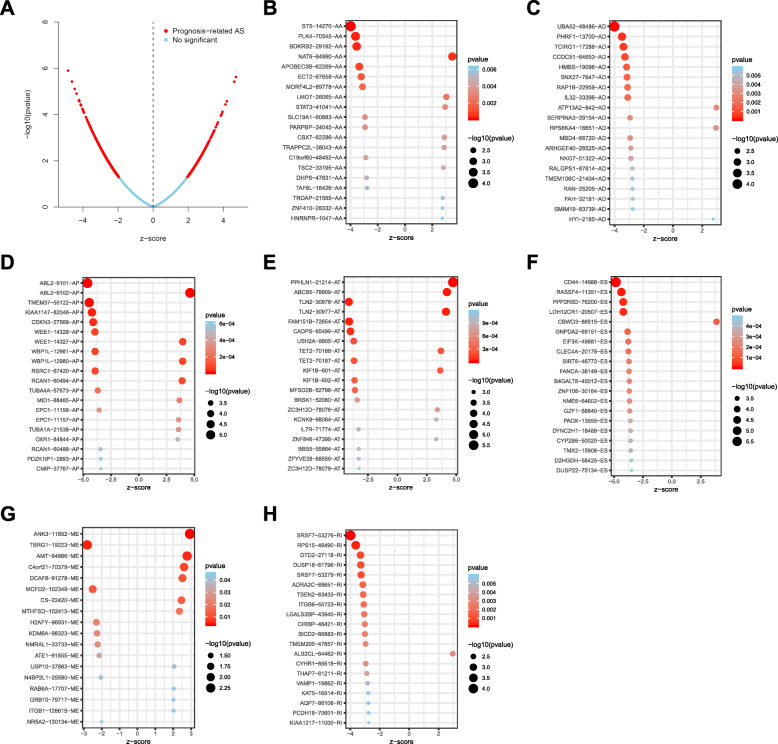
Top 20 most significant alternative splicing (AS) events in STAD. **a** Volcano plot of AS events. Each dot represents an AS event that occurs in a gene. The red dots represent AS events that are significantly correlated with patient survival. The blue dots represent AS events without correlation. Both z-score and *p*-value are statistical values generated by the previous univariate COX analysis, and they have a corresponding relationship (that is, the *p-*value can be obtained by searching the table according to z-score). Z-score represents Wald statistic, z-score > 0 corresponds to high risk AS events, z-score < 0 corresponds to low risk AS events. So all dots form a parabola finally. The top 20 AS events correlated with clinical outcome based on acceptor sites (**b**), alternate donor sites (**c**), alternate promoters (**d**), alternate terminators (**e**), exon skips (**f**), mutually exclusive exons (**g**), and retained introns (**h**)

**Fig. 3:**
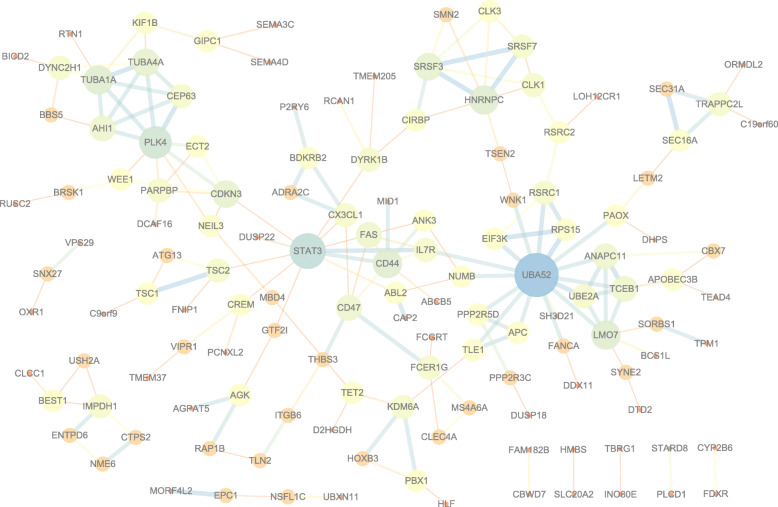
Protein-protein interaction network of genes with survival-associated alternative splicing events in STAD. For nodes, low degrees correspond to small sizes and bright colors, and high degrees correspond to large sizes and dark colors; For edges, low combined_scores correspond to small sizes and bright colors, and high combined_scores correspond to large sizes and dark colors

**Fig. 4:**
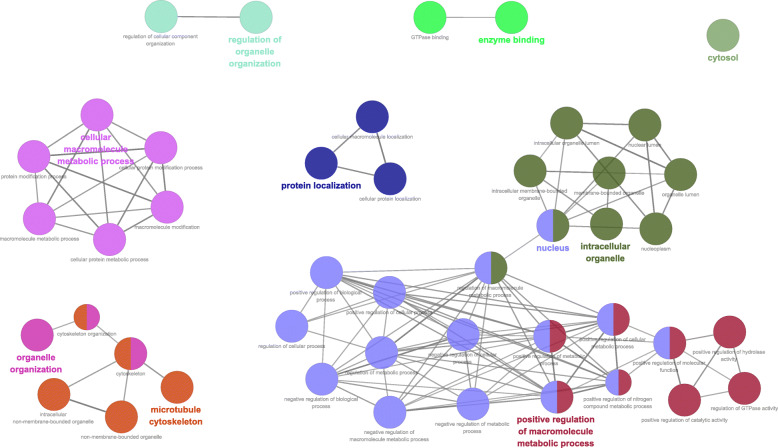
Cluego analysis for GO terminology (show only pathways with *P*-value ≤0.001). And each node represents a GO term, each line reflects the correlation between the terms, and the color embodies the function enrichment classification of the nodes, with same function aggregating together in same color

The distributions of AS events significantly correlated with patient survival are displayed in Fig. 2a. The 20 most significant prognosis-related AS events are also shown (Fig. 2b-h). To reveal the molecular characteristics of genes with survival-associated AS events, several bioinformatics analyses were conducted. First, a PPI network was constructed to demonstrate the relationships among these genes. UBA52, STAT3 and PLK4 ranked at the core in the network (Fig. 3). According to the functional annotations, “organelle organization”, “positive regulation of cellular process” and “protein localization” were the three most significant biological process terms. “intracellular organelle”, “membrane-bounded organelle” and “intracellular membrane-bounded organelle” were the three most significant cellular component terms. For molecular function, “enzyme binding” and “GTPase binding” were two most enriched categories (Fig. 4).

### Prognostic signatures for STAD patients

**Fig. 5:**
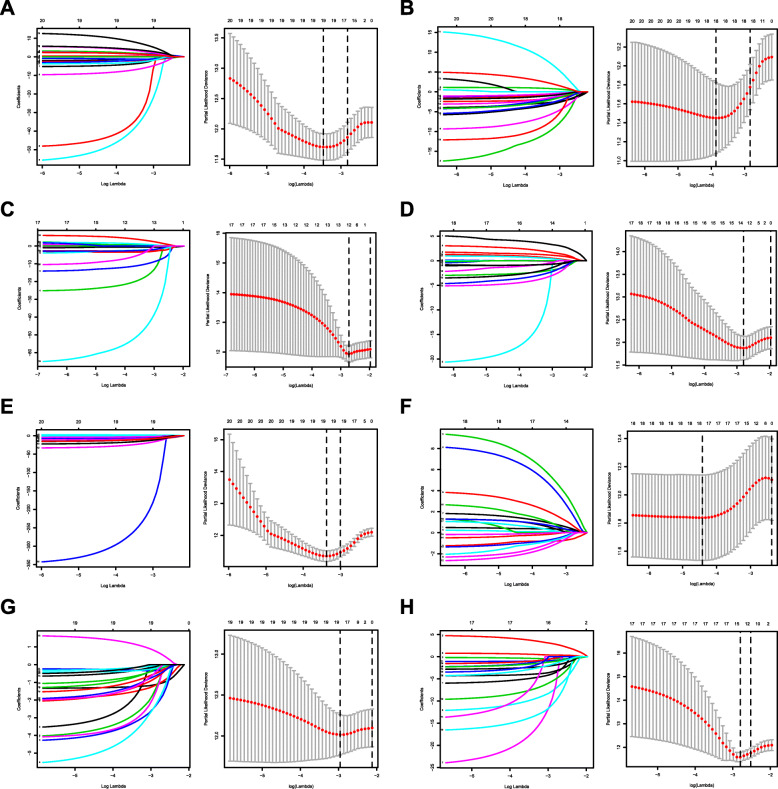
Construction of prognostic signatures based on LASSO COX analysis. Each right subfigure shows the Log Lambda value corresponding to the minimum cross-validation error point. And the AS events with non-zero coefficient corresponding to the same Log Lambda value were selected in the left figure for subsequent model construction. **a** Alternate Acceptor site (AA); **b** Alternate Donor site (AD); **c** Alternate Promoter (AP); **d** Alternate Terminator (AT); **e** Exon Skip (ES); **f** Mutually Exclusive Exons (ME); **g** Retained Intron (RI); and **h** All types of AS

**Fig. 6:**
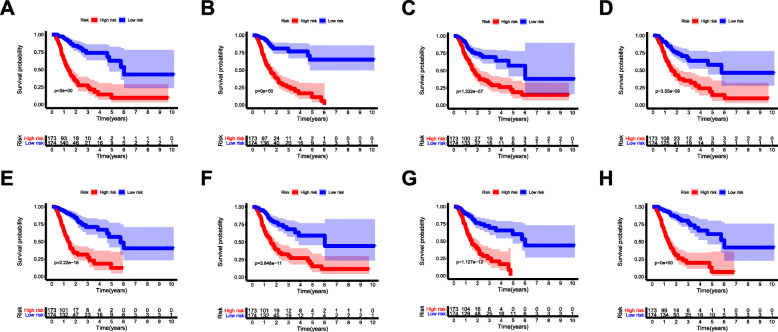
Kaplan-Meier curves of prognostic predictors for STAD. The lighter blue and red regions of each subfigure are the range of fluctuations, meaning 95%CI of the curve. **a** Alternate Acceptor site (AA); **b** Alternate Donor site (AD); **c** Alternate Promoter (AP); **d** Alternate Terminator (AT); **e** Exon Skip (ES); **f** Mutually Exclusive Exons (ME); **g** Retained Intron (RI); and **h** All types of AS

**Fig. 7:**
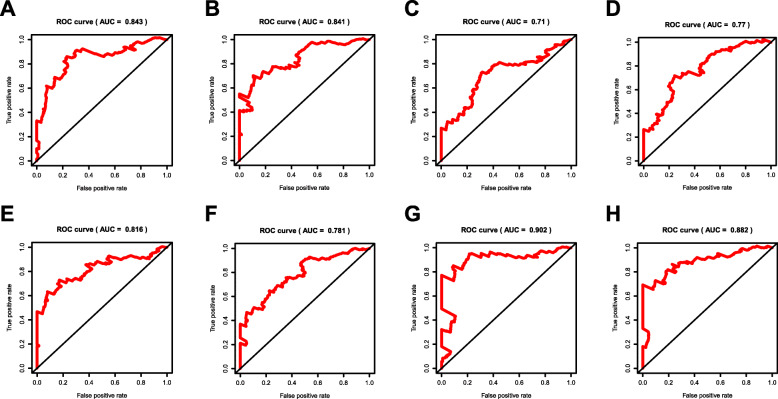
ROC curves of prognostic predictors for STAD. **a** Alternate Acceptor site (AA); **b** Alternate Donor site (AD); **c** Alternate Promoter (AP); **d** Alternate Terminator (AT); **e** Exon Skip (ES); **f** Mutually Exclusive Exons (ME); **g** Retained Intron (RI); and **h** All types of AS

**Fig. 8:**
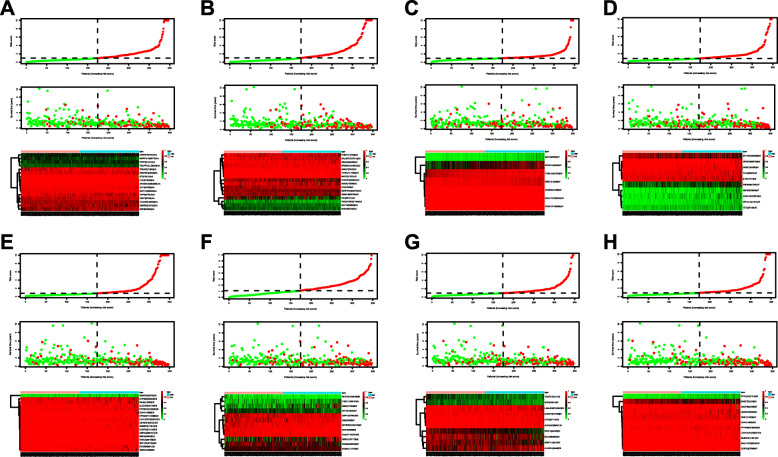
Determination and analysis of the prognostic AS signatures in the STAD cohort. STAD patients were divided into high- and low-risk subgroups based on the median cut of the risk score calculated separately. The upper part of each assembly represents the risk score curve (low risk patients are represented by green dots, high risk patients by red dots, and dash lines correspond to the median of all samples riskScore). The middle section represents the distribution of survival status and survival time of patients ranked by risk score (more green dots on the left for low-risk patients, and more red dots on the right for high-risk patients. From left to right, with the increase of the risk value, more and more patients died, indicating that the risk value is related to survival). The bottom heatmap displays the splicing pattern of the AS signature from each AS type or all seven AS types (the color transition from green to red indicates that the PSI value of the corresponding AS event increases from low to high). **a** Alternate Acceptor site (AA); **b** Alternate Donor site (AD); **c** Alternate Promoter (AP); **d** Alternate Terminator (AT); **e** Exon Skip (ES); **f** Mutually Exclusive Exons (ME); **g** Retained Intron (RI); and **h** All types of AS

**Fig. 9:**
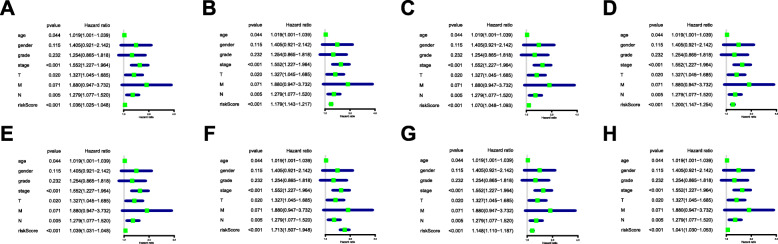
Univariate Cox regression analysis of clinical parameters and riskScore in STAD. For a clinical parameter or riskScore, if the *P*-value is less than 0.05, it is related to survival; if Hazard ratio is greater than 1, it is a high-risk factor. **a** Alternate Acceptor site (AA); **b** Alternate Donor site (AD); **c** Alternate Promoter (AP); **d** Alternate Terminator (AT); **e** Exon Skip (ES); **f** Mutually Exclusive Exons (ME); **g** Retained Intron (RI); and **h** All types of AS

**Fig. 10:**
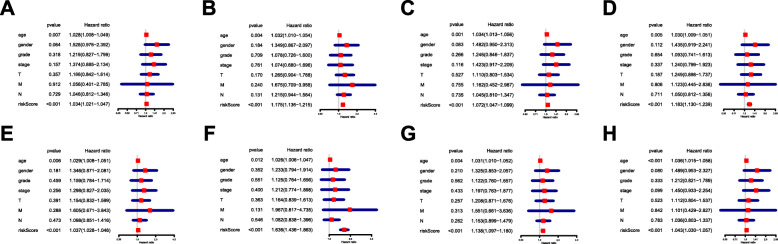
Multivariate Cox regression analysis of clinical parameters and riskScore in STAD. For the results of univariate and multivariate independent prognostic analysis, if the riskScore *P-*value of both is less than 0.05, it indicates that riskScore is independent of other clinical parameters and can be used as an independent prognostic factor in clinical practice. **a** Alternate Acceptor site (AA); **b** Alternate Donor site (AD); **c** Alternate Promoter (AP); **d** Alternate Terminator (AT); **e** Exon Skip (ES); **f** Mutually Exclusive Exons (ME); **g** Retained Intron (RI); and **h** All types of AS

By applying the LASSO Cox analysis following univariate Cox, which aims to filter out redundant genes and prevent overfitting of the model, we developed seven types of optimal prognostic signatures based on AA, AD, AP, AT, ES, ME and RI (Fig. 5, Table 1). According to Fig. 6, the eight models we constructed were able to separate the high and low risk groups well, because the differences between the high and low risk groups were very significant (although there were some overlaps of confidence intervals in the several subfigures, the overlaps were less, and the *p* values were very small, and the differences were significant). Therefore, the eight models could be used to predict the clinical results of STAD patients in clinical practice (Fig. 6). Eight ROC curves validated the performance of prognostic signatures in prognosis prediction, and their AUC values were all greater than 0.7, indicating that these eight models had certain accuracy (Fig. 7). Figure 8 shows the patient’s survival status and risk score, as well as the splicing pattern of AS signatures for each AS type or a combination of seven AS types. The upper risk score curve classified patients with low and high risk. The middle survival status figure indicated that the risk value was related to survival; although the decline of survival time was not obvious, the survival status was different. The bottom heat map shows the PSI value change of AS events with the increase of the risk value, in which if the PSI value of an AS event increases with the risk value, it indicated that the AS event was a high-risk AS event (Fig. 8). In univariate Cox analysis, the eight riskScores we constructed were all correlated with prognosis and high-risk factors (Fig. 9). According to multivariate independent prognostic analysis, riskScores obtained by the eight models could be used as independent prognostic factors, and all of them were high-risk factors (Fig. 10).

**Table 1:** Prognostic signatures for STAD

Type	Formula	Hazard ratio (95%CI)	AUC
AA	ST5–14270-AA*(− 5.351641) + PLK4–70545-AA*(− 50.075071) + BDKRB2–29192-AA*(− 2.251191) + NAT6–64990-AA*2.558247 + APOBEC3B-62,269-AA*(− 58.382634) + ECT2–67658-AA*(− 9.149068) + MORF4L2–89,778-AA*(− 2.400196) + STAT3–41041-AA*3.052117 + PARPBP-24045-AA*(− 3.451740) + CBX7–62286-AA*5.771950 + TRAPPC2L-38,043-AA*5.892537 + C19orf60–48,492-AA*(− 2.837545) + DHPS-47831-AA*(− 3.486833) + TROAP-21565-AA*2.928574 + ZNF410–28332-AA*13.901426 + HNRNPR-1047-AA*2.702840	1.034 (1.021–1.047)	0.843
AD	UBA52–48486-AD*(− 3.817624) + PHRF1–13700-AD*(− 2.535869) + TCIRG1–17286-AD*(− 15.912327) + CCDC51–64653-AD*(− 1.376775) + HMBS-19096-AD*(− 1.858043) + SNX27–7647-AD*(− 10.039140) + RAP1B-22,959-AD*(− 5.215765) + SERPINA3–29154-AD*(− 4.624829) + RPS6KA4–16,651-AD*14.672505 + MBD4–66720-AD*(− 3.903928) + NKG7–51322-AD*(− 13.306358) + RALGPS1–87614-AD*(− 4.395804) + TMEM106C-21,404-AD*(− 5.264665) + FAH-32181-AD*(− 0.998076) + SMIM19–83739-AD*(− 1.958156) + HYI-2185-AD*5.019022	1.175 (1.136–1.215)	0.841
AP	KIAA1147–82046-AP*(− 26.932098) + CDKN3–27569-AP*(− 16.593603) + WEE1–14328-AP*(− 4.589803) + RCAN1–60494-AP*0.979582 + MID1–88465-AP*3.529023 + TUBA1A-21,538-AP*7.031309 + PDZK1IP1–2893-AP*(− 77.071138)	1.072 (1.047–1.099)	0.71
AT	PPHLN1–21214-AT*4.712066 + ABCB5–78909-AT*1.406777 + TLN2–30978-AT*(− 3.649182) + TET2–70188-AT*2.736038 + MFSD2B-52,798-AT*(− 4.976167) + BRSK1–52060-AT*(− 3.402968) + ZC3H12D-78,076-AT*1.091190 + IL7R-71,774-AT*(− 4.704211) + ZNF846–47399-AT*1.093000 + ZFYVE28–68559-AT*(− 1.498791)	1.183 (1.130–1.239)	0.77
ES	CD44–14986-ES*(− 5.431100) + RASSF4–11351-ES*(− 15.542342) + PPP2R5D-76,200-ES*(− 9.142504) + LOH12CR1–20,507-ES*(− 14.620773) + CBWD3–86515-ES*2.727266 + GNPDA2–69151-ES*(− 13.336665) + EIF3K-49,681-ES*(− 5.515884) + CLEC4A-20,178-ES*(− 4.701354) + FANCA-38149-ES*(− 378.778875) + ZNF106–30164-ES*(− 34.393642) + NME6–64602-ES*(− 23.597673) + PAOX-13555-ES*(− 2.845972) + DYNC2H1–18,489-ES*(− 7.577542) + CYP2B6–50,020-ES*(− 1.561095) + TMX2–15906-ES*(− 4.527987) + D2HGDH-58,425-ES*(− 16.861342) + DUSP22–75134-ES*(− 16.679166)	1.037 (1.028–1.046)	0.816
ME	ANK3–11852-ME*0.617693 + AMT-64866-ME*9.363879 + C4orf21–70,379-ME*8.722888 + MCFD2–102349-ME*(− 2.786243) + CS-22420-ME*1.961533 + MTHFSD-102413-ME*3.738460 + KDM6A-98,323-ME*(− 1.285022) + ATE1–91855-ME*(− 2.737012) + USP10–37863-ME*1.511545 + RAB6A-17,707-ME*2.671001 + GRB10–79717-ME*1.261053 + ITGB1–126615-ME*1.273771	1.636 (1.436–1.863)	0.781
RI	SRSF7–53276-RI*(− 1.721712) + RPS15–46490-RI*(− 2.387697) + DTD2–27118-RI*(− 6.362278) + DUSP18–61796-RI*(− 5.322993) + ADRA2C-68,651-RI*(− 5.026148) + LGALS3BP-43,940-RI*(− 2.549496) + BICD2–86883-RI*(− 1.137099) + ALS2CL-64,462-RI*1.664472 + THAP7–61211-RI*(− 1.872850) + KAT5–16914-RI*(− 1.868716)	1.138 (1.097–1.180)	0.902
All	CD44–14986-ES*(− 6.883160) + PPHLN1–21214-AT*5.396354 + RASSF4–11351-ES*(− 14.293402) + KIAA1147–82046-AP*(− 22.278949) + PPP2R5D-76,200-ES*(− 6.035515) + LOH12CR1–20,507-ES*(− 10.075293) + CDKN3–27569-AP*(− 18.653016) + UBA52–48486-AD*(− 3.222755) + CADPS-65499-AT*(− 1.957771) + SRSF7–53276-RI*(− 2.217781) + WEE1–14328-AP*(− 5.279043)	1.043 (1.030–1.057)	0.882

### Survival-associated SF-AS network

**Fig. 11:**
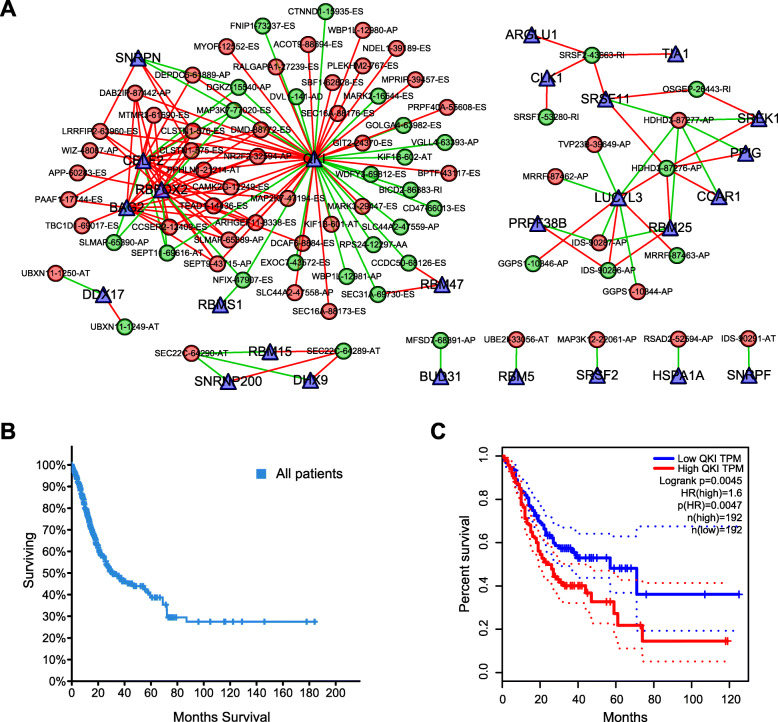
SF-AS network and survival analysis. **a** Survival-associated SF-AS network in STAD. AS is represented by circles (red for high-risk AS, green for low-risk AS), and SF is represented by triangles. A line between AS and SF indicates a regulatory relationship between them (the red line represents positive regulation and the green line represents negative regulation); **b** Overall survival of STAD patients; **c** Correlation analysis between the expression of QKI and the survival rate of STAD patients

Because events are primarily orchestrated by SFs that often bind with pre-mRNAs and regulate RNA splicing via influencing exon selection and splicing site. Therefore, exploration of the SF-AS regulatory network is imperative in STAD. Next, correlation analyses between the SFs’ expression and the most significant AS events’ PSI value (*P* < 0.001) were conducted (Fig. 11a). We observed that QKI was most significantly connected in the network, so we compared the influence of QKI expression on STAD’s survival rate. The consequence showed that low QKI expression significantly improved the survival rate of patients with STAD, and the five-year survival rate of the patients with low QKI expression was almost twice that of the patients with high QKI expression (Fig. 11b, Fig. 11c).

## Discussion

Currently, scientific research on the role of AS events in STAD still has many unanswered questions owing to the shortage of available large-sample public AS profiles and the paucity of systematic analysis referring to their clinical significance and deep molecular function. These bottlenecks have prevented cancer researchers from effectively recognizing the widespread applicability of AS events in STAD. Exploration of AS patterns broadens our vision and our understanding of traditional transcriptome molecular biomarkers. In this project, we adopted several biomedical computational approaches, which integrate the AS event profiles and clinical information of STAD patients to mine prognosis-related AS and construct splicing prognostic signatures that could stratify STAD patients into subgroups with distinct survival outcomes. Moreover, the SF-AS network could provide further insights into regulatory mechanisms in patients with STAD from the perspective of splicing.

Gastric cancer is a highly heterogeneous malignant tumor. Therefore, a single drug is not significantly useful for various types of gastric cancer. Classical cytotoxic therapy cannot be fully effective because of the presence of patients resistant to specific drugs. At present, the diagnosis and treatment of gastric cancer rely on histopathological diagnosis and definite classification. Therefore, in addition to targeted treatment with trastuzumab, we need to develop new targeted drugs to provide better treatment for patients. Potential biomarkers can be mined and used to predict patient outcomes, and treatment strategies can be developed for specific tumor parameters.

The next-generation sequencing technology developed in recent years adopts the whole- genome sequencing method, which has great advantages in exploring alternative splicing. Previously, several studies conducted SpliceSeq analyses to generate alterative splicing profiles for some types of cancer, as well as to construct prognostic signatures for cancer prognosis monitoring, including non-small cell lung cancer [15], colorectal cancer [16], and esophageal cancer [17]. This computational bioinformatics analysis could open up different perspectives on the clinical application and potential pathological mechanism of AS on a macro level. Previously, several studies have proposed transcriptomic signatures related to epithelial-to-mesenchymal transition and diagnosis of gastric cancer [18, 19]. The present in-depth study further explored alterations of transcriptomes used as prognostic predictors and could broaden our horizons in the clinical significance of transcriptomic signatures.

Given the multitude of AS events impacted by their own pre-mRNAs, the downstream functional impact is partly used to describe the molecular function of AS alteration events. In the PPI network analysis, UBA52, STAT3 and PLK4 were the hub genes. Previous studies have shown that UBA52 and STAT3 are all considered to be related molecules involved in the biological process of STAD. For example, bioinformatics analysis has verified the correlation between UBA52 and GC progress and metastasis [20]. STAT3 is a crucial transcription factor that regulates the transcription of many genes. It plays an extremely important role in promoting the occurrence and development of gastric cancer, and chronic STAT3 activation is a key event to induce the occurrence and development of gastric cancer [21]. STAT3 can directly up-regulate the epithelial expression of TLR2 in gastric tumors, which is related to the low survival rate of GC patients [22]. STAT3 signaling drives transcription activation of EZH2 and mediates poor prognosis in gastric cancer [23]. STAT3 promotes the increased expression of lncRNA HAGLROS, which leads to further progress of gastric cancer [24]. PLK4 is a serine/threonine protein kinase that regulates centriole duplication. Its maladjustment can lead to abnormal centrosomal numbers, mitotic defects, chromosomal instability, and ultimately tumorigenesis [25]. The relevant study has also shown that PLK4 overexpression in gastric cancer induces centrosome amplification and chromosomal instability, and leads to inhibition of primary cilia formation [26]. These findings also pave the way for future clinical applications, and related target inhibitors are being widely studied and clinically tested as new anticancer drugs. Functional enrichment analysis showed that in STAD, the main molecular function of AS event gene related to prognosis is to bind to GTPase, so it may provide selective advantages for cancer cells by regulating GTPase. Increased RhoA activity leads to poorer survival outcomes for the Lauren diffuse type of gastric adenocarcinoma (DGA), and inhibition of RhoA can correct the drug resistance of DGA [27]. RacGAP1 is closely associated with malignant progression and poor survival [28]. Leptin promotes GC migration through the Rho/ROCK mechanism [29]. RASSF6 partially regulates the effect of mir-181a-5p on GC progression through the MAKP pathway [30]. It is worth considering that, in gastric cancer cells, RhoA promotes cell proliferation and RhoC stimulates cell migration and invasion, while RhoB functions contrary to RhoA and/or RhoC [31]. Therefore, targeted GTPase therapy is also being explored. For example, ALEX1 functions in gastric cancer through the PAR-1/Rho GTPase signaling pathway, becoming a new target for tumor inhibition [32]. RhoA-mediated Fbxw7 regulates the apoptosis of tumor cells and other phenotypes in gastric cancer [33]. Similarly, Gastrokine 1’s inhibition of gastric cancer progression may also be dependent on RhoA [34]. Our findings suggest that a group of AS events play a biological role in the alteration of GTPase in STAD.

The highlight of the current study was that we proposed prognostic signatures based on AS events for monitoring the prognosis of STAD patients. Recently, some prognostic signatures in STAD have been proposed. Zhang H et al. found that the efficacy of postoperative adjuvant chemotherapy for gastric cancer was affected by the degree of neutrophil infiltration of the tumor [35]. Jiang Y et al. developed an immune score GC classifier that can effectively predict the recurrence and survival of patients with gastric cancer, which plays a good role in complementation of the prognosis judgment for the TNM staging system [36]. The clinical management of STAD patients still needs to be improved, and the above mentioned molecular biomarkers have broad prospects. In order to facilitate clinical practice, we selected a group of AS events using the LASSO Cox regression model, and the prognostic model proposed on which showed satisfactory results. However, our prognostic model has limitations because our work completely based on the bioinformatic analysis and lacked an independent validation cohort. In addition, in order to provide more explanation details, our study also needed a wet lab validation. Due to the limited public alternative splicing data currently, the sample size used to construct a prognostic model is small. If these samples are forcibly divided into a training group and a validation group, the sample size used to construct the prognostic model will be less, leading to poor accuracy of the prognostic model. Besides, on account of the Corona virus disease 2019 pandemic, our Wuhan laboratory has been closed for a long time, and there is no objective condition to collect clinical samples for genetic testing.

We believe that the TCGA data we used were appropriately standardized. However, the Lasso model adopts the square loss function and applies the same tuning parameter to all variables. Once outliers exist in the data, the estimator obtained is biased, resulting in poor robustness. Therefore, it may be a potential problem that leads to the imperfect accuracy of the prognostic model. Robust analysis methods using outliers to process data for high-dimensional genetic data analysis have been developed and are rapidly gaining popularity. Among them, LAD (least absolute deviation) –LASSO is a method to combine the regression shrinkage and selection of LASSO and robustness of LAD for outliers and heavy-tailed errors [37, 38]. In theory, the test results may be more meaningful through robust variable selection.

A large number of AS events are programmed by finite SFs in cells [39]. The altered profile of AS events in multiple tumor types emphasizes the important mechanism of splicing factors in cancer, which is disordered splicing [40]. It is increasingly believed that changes of SFs in STAD can be involved in tumorigenesis and progression through various mechanisms [41–43]. The splicing correlation network analysis has also found out the larger regulated nodes, indicating that they occupy a significant position in the SF-AS network. QKI, which is recognized as a tumor suppressor in a wide range of cancers, is highly connected in the network, which can play a significant part in the prognosis induced by splicing events [44–46]. Multiple-factor analysis of a related study shows that QKI expression is an independent prognostic factor for the survival of GC patients [47]. But the role of QKI in STAD has not been fully discussed yet. Our study indicates that the level of QKI expression is significantly correlated with the survival rate of patients with STAD, and it can become an important target for drug design in the future. Nevertheless, our algorithm suggested deregulated AS events as a hallmark of STAD. However, there are some limitations inevitably affecting the reliability of the study. Firstly, we didn’t use a separate cohort for more validation. Secondly, more functional experiments are needed to further investigate the impact of dysregulated AS events and SFs on carcinogenesis.

## Conclusions

In conclusion, the current study has found out a phenomenological relationship between AS events and prognosis in STAD patients, which is the base of unscrambling the functional contribution of AS events in STAD. These findings are conducive to develop new genomic models for clinical cancer management. In addition, the further identification of predictive splicing factors for prognosis and the construction of SF-AS networks will pave the way for further exploration of splicing related mechanisms.

## Methods

### Data acquisition

TCGA SpliceSeq [48] is a data portal that provides AS profiles across 33 tumors based on TCGA RNA-seq data. SpliceSeq evaluates seven types of splice events, including alternate acceptor (AA), alternate donor (AD), alternate promoter (AP), alternate terminator (AT), exon skip (ES), mutually exclusive exon (ME) and retained intron (RI). TCGA SpliceSeq processed the percent spliced in (PSI) value for cancer research analysis, which indicates the inclusion of a transcript element divided by the total number of reads for that AS event. Alterations in PSI values range from 0 to 100 (%), which suggests a shift in splicing events. The filtering condition for downloading data from the TCGA SpliceSeq website was the percentage of samples with PSI value ≥75. The AS events with standard diversion < 1 were removed.

Clinical information of STAD patients was also obtained from the TCGA database. Only pathologically confirmed STAD patients with both follow-up and AS event data were included for our analysis. For clinical information downloaded from TCGA, we deleted patients with survival time < 90 days or null data, and included a total of 338 patients (Table 2) for subsequent analysis. For advanced cancer patients with survival time < 90 days (usually along with severe metabolic disorders such as cachexia and endotoxin), the effect of alternative splicing events in promoting cancer development is no longer accurate, so patients with survival time < 90 days were removed. The same TCGA ID was used to integrate clinical information and AS events data.

**Table 2:** Clinical characteristics of STAD patients in the TCGA database

Characteristics		Total	%
All		338	100.00
Age (y)	≥65	189	55.92
	< 65	149	44.08
Gender	Male	216	63.91
	Female	122	36.09
Grade	G1	7	2.07
	G2	116	34.32
	G3	215	63.61
	G4	0	0.00
Stage	I	42	12.43
	II	112	33.14
	III	153	45.27
	IV	31	9.17
T stage	T1	15	4.44
	T2	72	21.30
	T3	167	49.41
	T4	84	24.85
M stage	M0	319	94.38
	M1	19	5.62
N stage	N0	104	30.77
	N1	95	28.11
	N2	71	21.01
	N3	68	20.12

### Survival analysis

In the survival analysis, the follow-up periods ranged from 90 days to 3720 days after removal of patients with survival less than 90 days. Univariate Cox analysis was conducted to assess the correlations between the PSI value (from 0 to 100) of each AS event and the survival data of STAD patients (*P* < 0.05). We input the corresponding genes into the Search Tool for the Retrieval of Interacting Genes (STRING) database [49], and the constructed protein-protein interaction (PPI) network was adjusted by Cytoscape software [50]. Meanwhile, we applied the ClueGO plug-in [51] in Cytoscape software for Gene Ontology (GO) and Kyoto Encyclopedia of Genes and Genomes (KEGG) enrichment analysis and drew the enrichment network. The least absolute shrinkage and selection operator (LASSO) method is a widely used regression analysis method of high-dimensional predictors [52]. LASSO has been extended for use in Cox regression survival analysis and is ideal for high- dimensional data. We selected the LASSO Cox regression model to determine the accurate coefficient for each prognostic feature and to estimate the deviance likelihood via 1-standard error (SE) criteria. The coefficients and partial likelihood deviance were calculated with the “glmnet” package in R.

### Prognostic signature construction

The significant AS events in univariate Cox analysis were submitted to LASSO regression analysis to develop prognostic signatures based on seven types of AS events. Finally, prognostic signatures for survival prediction were calculated by multiplying the PSI values of prognostic indictors and the coefficient assigned by LASSO regression analysis. The riskScore of each patient was calculated according to the constructed prognostic signatures. Based on the median value of all patients’ riskScores, all patients were divided into high and low risk groups. Then, survival analysis was carried out for the high and low risk groups to obtain the *P*-values of survival difference and survival curves. The ROC curve was plotted using the survivalROC package, primarily to determine the accuracy of the prognostic model. By incorporating the following parameters into multivariate Cox regression analysis, splicing-based prognostic signature was evaluated as independent predictors: age, gender, grade, stage, TMN stage.

### SF-AS regulatory network

A compendium of 404 splicing factors was obtained from a previous study [53]. The expression profiles of SF genes were curated from the TCGA dataset. We selected axes between the expression value of SFs and PSI values of prognosis-related AS events to construct the SF-AS regulatory network according to the following conditions: *P* value less than 0.001 and the absolute value of Pearson’s correlation coefficient more than 0.6. Then, we built the correlation plots via Cytoscape version 3.7.1. All R code and annotations have also been submitted (Additional file 3).

## Supplementary information

**Additional file 1.** The initial clinical data downloaded from the TCGA website is in the file Additional file 1.

**Additional file 2.** The file Additional file 2 contains all surviving-related AS events.

**Additional file 3.** All R code and annotations in the file Additional file 3 have also been submitted.

## Data Availability

Gene expression data and clinical information of STAD were publicly available in The Cancer Genome Atlas (TCGA) database (https://portal.gdc.cancer.gov/repository?facetTab=cases). In the left box, select TCGA-STAD and HTSeq - FPKM, add all files to Cart, and then Gene expression data of STAD can be downloaded; select TCGA-STAD, clinical and bcr xml in turn, add all files to Cart, and then clinical information of STAD can be downloaded. Alternative splicing data of STAD were downloaded from TCGA SpliceSeq website (http://projects.insilico.us.com/TCGASpliceSeq/PSIdownload.jsp). Select the Stomach Adenocarcinoma [STAD], all Splice Event Types in turn, and then the Alternative splicing data of STAD can be downloaded.

## Abbreviations

AS: Alternative splicing
STAD: Stomach adenocarcinoma
TCGA: The Cancer Genome Atlas
SF: Splicing factor
AUC: Area under the curve
ROC: Receiver operating characteristic
GC: Gastric cancer
AA: Alternate acceptor
AD: Alternate donor
AP: Alternate promoter
AT: Alternate terminator
ES: Exon skip
ME: Mutually exclusive exon
RI: Retained intron
PSI: Percent spliced in
STRING: Search Tool for the Retrieval of Interacting Genes
PPI: Protein-protein interaction
GO: Gene Ontology
KEGG: Kyoto Encyclopedia of Genes and Genomes
LASSO: The least absolute shrinkage and selection operator
SE: Standard error
DGA: The Lauren diffuse type of gastric adenocarcinoma
LAD: Least absolute deviation

## References

[1] Jemal A, Bray F, Center MM, Ferlay J, Ward E, Forman D (2011). Global cancer statistics. CA Cancer J Clin.

[2] Cutsem EV, Sagaert X, Topal B, Haustermans K, Prenen H (2016). Gastric cancer. Lancet.

[3] Van Cutsem E, Dicato M, Geva R, Arber N, Bang Y, Benson A, Cervantes A, Diaz-Rubio E, Ducreux M, Glynne-Jones R (2011). The diagnosis and management of gastric cancer: expert discussion and recommendations from the 12th ESMO/world congress on gastrointestinal Cancer, Barcelona, 2010. Ann Oncol.

[4] Lutz MP, Zalcberg JR, Ducreux M, Ajani JA, Allum W, Aust D, Bang YJ, Cascinu S, Holscher A, Jankowski J (2012). Highlights of the EORTC St. Gallen international expert consensus on the primary therapy of gastric, gastroesophageal and oesophageal cancer - differential treatment strategies for subtypes of early gastroesophageal cancer. Eur J Cancer.

[5] Nilsen TW, Graveley BR (2010). Expansion of the eukaryotic proteome by alternative splicing. Nature.

[6] Wang ET, Sandberg R, Luo S, Khrebtukova I, Zhang L, Mayr C, Kingsmore SF, Schroth GP, Burge CB (2008). Alternative isoform regulation in human tissue transcriptomes. Nature.

[7] Salton M, Misteli T (2016). Small molecule modulators of pre-mRNA splicing in Cancer therapy. Trends Mol Med.

[8] Wahl MC, Will CL, Luhrmann R (2009). The spliceosome: design principles of a dynamic RNP machine. Cell.

[9] Ge Y, Porse BT (2014). The functional consequences of intron retention: alternative splicing coupled to NMD as a regulator of gene expression. Bioessays.

[10] Song X, Zeng Z, Wei H, Wang Z (2018). Alternative splicing in cancers: from aberrant regulation to new therapeutics. Semin Cell Dev Biol.

[11] David CJ, Manley JL (2010). Alternative pre-mRNA splicing regulation in cancer: pathways and programs unhinged. Genes Dev.

[12] Oltean S, Bates DO (2014). Hallmarks of alternative splicing in cancer. Oncogene.

[13] Sveen A, Kilpinen S, Ruusulehto A, Lothe RA, Skotheim RI (2016). Aberrant RNA splicing in cancer; expression changes and driver mutations of splicing factor genes. Oncogene.

[14] Ladomery M (2013). Aberrant alternative splicing is another hallmark of cancer. Int J Cell Biol.

[15] Li Y, Sun N, Lu Z, Sun S, Huang J, Chen Z, He J (2017). Prognostic alternative mRNA splicing signature in non-small cell lung cancer. Cancer Lett.

[16] Xiong Y, Deng Y, Wang K, Zhou H, Zheng X, Si L, Fu Z (2018). Profiles of alternative splicing in colorectal cancer and their clinical significance: a study based on large-scale sequencing data. EBioMedicine.

[17] Mao S, Li Y, Lu Z, Che Y, Sun S, Huang J, Lei Y, Wang X, Liu C, Zheng S (2019). Survival-associated alternative splicing signatures in esophageal carcinoma. Carcinogenesis.

[18] Xu B, Bai Z, Yin J, Zhang Z (2019). Global transcriptomic analysis identifies SERPINE1 as a prognostic biomarker associated with epithelial-to-mesenchymal transition in gastric cancer. PeerJ.

[19] Cui J, Chen Y, Chou WC, Sun L, Chen L, Suo J, Ni Z, Zhang M, Kong X, Hoffman LL (2011). An integrated transcriptomic and computational analysis for biomarker identification in gastric cancer. Nucleic Acids Res.

[20] Tian X, Ju H, Yang W (2019). An ego network analysis approach identified important biomarkers with an association to progression and metastasis of gastric cancer. J Cell Biochem.

[21] G. AS, M. TR, J. LM (2012). Targeting STAT3 in gastric cancer. Expert Opin Ther Targets.

[22] Tye H, Kennedy Catherine L, Najdovska M, McLeod L, McCormack W, Hughes N, Dev A, Sievert W, Ooi Chia H, Ishikawa T-o (2012). STAT3-driven Upregulation of TLR2 promotes gastric tumorigenesis independent of tumor inflammation. Cancer Cell.

[23] Pan YM, Wang CG, Zhu M, Xing R, Cui JT, Li WM, Yu DD, Wang SB, Zhu W, Ye YJ (2016). STAT3 signaling drives EZH2 transcriptional activation and mediates poor prognosis in gastric cancer. Mol Cancer.

[24] Chen JF, Wu P, Xia R, Yang J, Huo XY, Gu DY, Tang CJ, De W, Yang F (2018). STAT3-induced lncRNA HAGLROS overexpression contributes to the malignant progression of gastric cancer cells via mTOR signal-mediated inhibition of autophagy. Mol Cancer.

[25] Zhao Y, Wang X (2019). PLK4: a promising target for cancer therapy. J Cancer Res Clin Oncol.

[26] Shinmura K, Kurabe N, Goto M, Yamada H, Natsume H, Konno H, Sugimura H (2014). PLK4 overexpression and its effect on centrosome regulation and chromosome stability in human gastric cancer. Mol Biol Rep.

[27] Yoon C, Cho SJ, Aksoy BA, Park DJ, Schultz N, Ryeom SW, Yoon SS (2016). Chemotherapy resistance in diffuse-type gastric adenocarcinoma is mediated by RhoA activation in Cancer stem-like cells. Clin Cancer Res.

[28] Saigusa S, Tanaka K, Mohri Y, Ohi M, Shimura T, Kitajima T, Kondo S, Okugawa Y, Toiyama Y, Inoue Y (2015). Clinical significance of RacGAP1 expression at the invasive front of gastric cancer. Gastric Cancer.

[29] Dong Z, Fu S, Xu X, Yang Y, Du L, Li W, Kan S, Li Z, Zhang X, Wang L (2014). Leptin-mediated regulation of ICAM-1 is rho/ROCK dependent and enhances gastric cancer cell migration. Br J Cancer.

[30] Mi Y, Zhang D, Jiang W, Weng J, Zhou C, Huang K, Tang H, Yu Y, Liu X, Cui W (2017). miR-181a-5p promotes the progression of gastric cancer via RASSF6-mediated MAPK signalling activation. Cancer Lett.

[31] Zhou J, Zhu Y, Zhang G, Liu N, Sun L, Liu M, Qiu M, Luo D, Tang Q, Liao Z (2011). A distinct role of RhoB in gastric cancer suppression. Int J Cancer.

[32] Pang L, Li JF, Su L, Zang M, Fan Z, Yu B, Wu X, Li C, Yan M, Zhu ZG (2018). ALEX1, a novel tumor suppressor gene, inhibits gastric cancer metastasis via the PAR-1/rho GTPase signaling pathway. J Gastroenterol.

[33] Li H, Wang Z, Zhang W, Qian K, Xu W, Zhang S (2016). Fbxw7 regulates tumor apoptosis, growth arrest and the epithelial-to-mesenchymal transition in part through the RhoA signaling pathway in gastric cancer. Cancer Lett.

[34] Yoon JH, Choi WS, Kim O, Choi BJ, Nam SW, Lee JY, Park WS (2017). Gastrokine 1 inhibits gastric cancer cell migration and invasion by downregulating RhoA expression. Gastric Cancer.

[35] Zhang H, Liu H, Shen Z, Lin C, Wang X, Qin J, Qin X, Xu J, Sun Y (2018). Tumor-infiltrating neutrophils is prognostic and predictive for postoperative adjuvant chemotherapy benefit in patients with gastric Cancer. Ann Surg.

[36] Jiang Y, Zhang Q, Hu Y, Li T, Yu J, Zhao L, Ye G, Deng H, Mou T, Cai S (2018). ImmunoScore signature: a prognostic and predictive tool in gastric Cancer. Ann Surg.

[37] Wu C, Ma S (2015). A selective review of robust variable selection with applications in bioinformatics. Brief Bioinform.

[38] Ren J, Du Y, Li S, Ma S, Jiang Y, Wu C (2019). Robust network-based regularization and variable selection for high-dimensional genomic data in cancer prognosis. Genet Epidemiol.

[39] Lee Y, Rio DC (2015). Mechanisms and regulation of alternative pre-mRNA splicing. Annu Rev Biochem.

[40] Zhang J, Manley JL (2013). Misregulation of pre-mRNA alternative splicing in cancer. Cancer Discov.

[41] Zhu S, Chen Z, Katsha A, Hong J, Belkhiri A, El-Rifai W (2016). Regulation of CD44E by DARPP-32-dependent activation of SRp20 splicing factor in gastric tumorigenesis. Oncogene.

[42] Butkyte S, Ciupas L, Jakubauskiene E, Vilys L, Mocevicius P, Kanopka A, Vilkaitis G (2016). Splicing-dependent expression of microRNAs of mirtron origin in human digestive and excretory system cancer cells. Clin Epigenetics.

[43] Park WC, Kim HR, Kang DB, Ryu JS, Choi KH, Lee GO, Yun KJ, Kim KY, Park R, Yoon KH (2016). Comparative expression patterns and diagnostic efficacies of SR splicing factors and HNRNPA1 in gastric and colorectal cancer. BMC Cancer.

[44] Danan-Gotthold M, Golan-Gerstl R, Eisenberg E, Meir K, Karni R, Levanon EY (2015). Identification of recurrent regulated alternative splicing events across human solid tumors. Nucleic Acids Res.

[45] Chen AJ, Paik JH, Zhang H, Shukla SA, Mortensen R, Hu J, Ying H, Hu B, Hurt J, Farny N (2012). STAR RNA-binding protein quaking suppresses cancer via stabilization of specific miRNA. Genes Dev.

[46] Zong FY, Fu X, Wei WJ, Luo YG, Heiner M, Cao LJ, Fang Z, Fang R, Lu D, Ji H (2014). The RNA-binding protein QKI suppresses cancer-associated aberrant splicing. PLoS Genet.

[47] Bian Y, Wang L, Lu H, Yang G, Zhang Z, Fu H, Lu X, Wei M, Sun J, Zhao Q (2012). Downregulation of tumor suppressor QKI in gastric cancer and its implication in cancer prognosis. Biochem Biophys Res Commun.

[48] Ryan M, Wong WC, Brown R, Akbani R, Su X, Broom B, Melott J, Weinstein J (2016). TCGASpliceSeq a compendium of alternative mRNA splicing in cancer. Nucleic Acids Res.

[49] Szklarczyk D, Morris JH, Cook H, Kuhn M, Wyder S, Simonovic M, Santos A, Doncheva NT, Roth A, Bork P (2017). The STRING database in 2017: quality-controlled protein-protein association networks, made broadly accessible. Nucleic Acids Res.

[50] Su G, Morris JH, Demchak B, Bader GD (2014). Biological network exploration with Cytoscape 3. Curr Protoc Bioinformatics.

[51] Bindea G, Mlecnik B, Hackl H, Charoentong P, Tosolini M, Kirilovsky A, Fridman WH, Pages F, Trajanoski Z, Galon J (2009). ClueGO: a Cytoscape plug-in to decipher functionally grouped gene ontology and pathway annotation networks. Bioinformatics.

[52] Tibshirani R (1997). The lasso method for variable selection in the cox model. Stat Med.

[53] Seiler M, Peng S, Agrawal AA, Palacino J, Teng T, Zhu P, Smith PG (2018). Cancer genome atlas research N, Buonamici S, Yu L: somatic mutational landscape of splicing factor genes and their functional consequences across 33 Cancer types. Cell Rep.

